# Climate-responsive telemedicine: Innovative strategy for enhancing healthcare in the face of climate change

**DOI:** 10.7189/jogh.14.03043

**Published:** 2024-10-18

**Authors:** Muhammad Fawad, Saif Ullah, Xiaolin Xu

**Affiliations:** 1 School of Public Health, The Second Affiliated Hospital, Zhejiang University School of Medicine, Hangzhou, Zhejiang, China; 2 Department of Gastroenterology, The First Affiliated Hospital of Zhengzhou University, Zhengzhou, Henan, China

Climate change presents unique challenges to global health, especially for vulnerable populations. Although telemedicine has emerged as a vital tool for delivering healthcare, particularly during the coronavirus disease 2019 (COVID-19) pandemic, its potential in the context of climate change remains unclear. Here we introduce a novel approach called ‘climate responsive telemedicine’ (CRT), incorporating climate resilience into telemedicine services. Climate change is known to intensify health issues such as respiratory diseases, heat-related illnesses, and infectious diseases [1]. Telemedicine has shown significant potential for providing accessible healthcare [2], yet its vulnerability to climate-induced challenges is often overlooked. Such issues emerge from climate stress, where traditional telemedicine systems occasionally lack sufficient resilience to cope with severe weather conditions, which can cause significant disruptions in service delivery during critical times.

The CRT approach we propose here incorporates specific features designed to maintain functionality during climatic events, ensuring uninterrupted access to healthcare for the affected populations. Specifically, it provides a telemedicine system resilient to climate-induced disruptions by following three key areas. First, it emphasises the development of infrastructure capable of withstanding extreme weather events. This includes upgrading telecommunication networks, using weather-resistant materials in construction, implementing flood-resistant systems, and ensuring robust data protection. Overcoming technological obstacles, such as installing long-lasting solar panels and maintaining reliable satellite communication, is crucial for this process. Second, the system delivers climate-specific health services, including teleconsultations during heatwaves or pollution spikes, modification of service protocols, training healthcare professionals to effectively address health issues related to climate change, and educating patients on maintaining their health during extreme weather conditions. This includes guidance on effective hydration techniques and the early detection of climate-related illnesses. Lastly, to ensure access in climate-vulnerable communities, the system focuses on cost-effective and scalable solutions by integrating solar-powered devices and satellite communication to maintain connectivity. The deployment of these technologies presents technical and logistical challenges, such as ensuring the resilience of solar panels in severe weather conditions and the reliability of satellite communication during storms. Tailoring health services to address climate-specific health risks and collaborating with local healthcare providers and policymakers to align with existing health systems and climate policies is essential.

We propose conducting pilot studies in diverse geographical areas, particularly those highly vulnerable to climate change, to evaluate the feasibility, effectiveness, and community impact of CRT. These geographical areas should be selected based on factors such as historical climate data, the prevalence of climate-sensitive health issues, and the accessibility of telecommunication infrastructure. The pilot studies will use robust techniques, including randomised controlled trials and longitudinal assessments, to assess the adaptability of CRT to various climate challenges and its effectiveness in improving health outcomes in communities during extreme weather events. Meanwhile, health outcomes, patient satisfaction, and system reliability are among the specific metrics used to assess the efficacy of the approach. While implementing these studies, potential challenges may arise, including resistance from the local community, technological failures, logistical difficulties in establishing pilot sites, and variations in the local climate conditions. They can be addressed by thorough planning, actively involving the community, closely collaborating with local stakeholders, using adaptable study designs, and establishing strong monitoring and assessment systems.

CRT offers a proactive approach that addresses immediate health needs during climatic events and contributes to the sustainability of long-term healthcare systems. For instance, during a heatwave in a rural area, it may enable remote consultations with healthcare specialists who can quickly offer advice on treating heat-related diseases. This may include counselling on hydration, cooling strategies, and identifying early signs of heatstroke. Additionally, CRT can help connect patients to local resources, such as emergency services or cooling facilities, reducing the need for hospitalisation and lowering the risk of fatalities. It ensures that vulnerable individuals receive appropriate health treatments promptly, even during extreme weather conditions. These findings align with insights from studies on healthcare sustainability and climate change [3], telemedicine implementation challenges [4], and integration of telemedicine into healthcare systems [5]. Furthermore, the focus of CRT on climate-specific health conditions is informed by telemedicine applications for managing health conditions like heart failure [6] and its effectiveness during public health crises, such as the COVID-19 pandemic [7].

CRT has distinct benefits over traditional telemedicine in terms of improved resistance to climate-related disruptions and the ability to address health risks specific to climate conditions. However, it may also have limitations, such as higher upfront costs and the requirement for specialised training for healthcare professionals.

Telemedicine has the potential to significantly reduce greenhouse gas emissions by minimising patient travel and limiting the operational footprint of healthcare facilities [8,9]. For instance, using energy-efficient technologies and reducing the need for physical travel can contribute to this reduction. CRT expands this concept by incorporating sustainable technologies, further enhancing its environmental benefits.

CRT represents a novel and necessary integration of telemedicine with climate change adaptation. As global health faces the dual challenges of climate change and access to healthcare, the approach offers a promising solution for ensuring resilient and equitable health service delivery. Its success, however, depends on the incorporation of policy support. Policymakers play an important role in integrating CRT into existing healthcare and climate policies by providing sufficient funding resources, establishing conducive regulatory platforms, and promoting cooperation among stakeholders. This involves coordinating CRT programmes with both national and international health and climate policies, promoting collaboration with local healthcare professionals, and ensuring that telemedicine infrastructure adheres to resilience requirements. Supporting active participation from international and local stakeholders could further improve the efficiency and expandability of CRT.

Future studies should focus on improving CRT infrastructure, comparing the findings of the pilot study, and exploring innovative technologies for adapting to climate change. We invite further discussion and collaboration in this emerging field to explore its potential for implementation, especially with stakeholders, such as policymakers, healthcare providers, and technology developers, who may participate in the advancement of CRT through cooperative platforms, seminars, and pilot projects aimed at improving and expanding CRT initiatives.
